# Boron Nitride Nanotube-Mediated Stimulation of Cell Co-Culture on Micro-Engineered Hydrogels

**DOI:** 10.1371/journal.pone.0071707

**Published:** 2013-08-14

**Authors:** Leonardo Ricotti, Toshinori Fujie, Helena Vazão, Gianni Ciofani, Roberto Marotta, Rosaria Brescia, Carlo Filippeschi, Irene Corradini, Michela Matteoli, Virgilio Mattoli, Lino Ferreira, Arianna Menciassi

**Affiliations:** 1 The BioRobotics Institute, Scuola Superiore Sant’Anna, Pontedera, Pisa, Italy; 2 Center of MicroBioRobotics @ SSSA, Istituto Italiano di Tecnologia, Pontedera, Pisa, Italy; 3 Biocant - Center of Biotechnology Innovation Center, Cantanhede, Coimbra, Portugal; 4 CNC – Center for Neuroscience and Cell Biology, University of Coimbra, Coimbra, Portugal; 5 WPI - Advanced Institute for Materials Research, Tohoku University, Sendai, Japan; 6 Fondazione Filarete, Milano, Italy; 7 Dipartimento di Biotecnologie Mediche e Medicina Traslazionale, Università degli Studi di Milano, Milano, Italy; 8 Humanitas Clinical and Research Center, Rozzano, Italy; 9 Istituto Italiano di Tecnologia, Genova, Italy; University of California, Merced, United States of America

## Abstract

In this paper, we describe the effects of the combination of topographical, mechanical, chemical and intracellular electrical stimuli on a co-culture of fibroblasts and skeletal muscle cells. The co-culture was anisotropically grown onto an engineered micro-grooved (10 µm-wide grooves) polyacrylamide substrate, showing a precisely tuned Young’s modulus (∼ 14 kPa) and a small thickness (∼ 12 µm). We enhanced the co-culture properties through intracellular stimulation produced by piezoelectric nanostructures (i.e., boron nitride nanotubes) activated by ultrasounds, thus exploiting the ability of boron nitride nanotubes to convert outer mechanical waves (such as ultrasounds) in intracellular electrical stimuli, by exploiting the direct piezoelectric effect. We demonstrated that nanotubes were internalized by muscle cells and localized in both early and late endosomes, while they were not internalized by the underneath fibroblast layer. Muscle cell differentiation benefited from the synergic combination of topographical, mechanical, chemical and nanoparticle-based stimuli, showing good myotube development and alignment towards a preferential direction, as well as high expression of genes encoding key proteins for muscle contraction (i.e., actin and myosin). We also clarified the possible role of fibroblasts in this process, highlighting their response to the above mentioned physical stimuli in terms of gene expression and cytokine production. Finally, calcium imaging-based experiments demonstrated a higher functionality of the stimulated co-cultures.

## Introduction

The achievement of mature and functional *in vitro* engineered skeletal muscle constructs is a challenge that could open new horizons in different fields. Regenerative medicine-oriented applications represent an important motivation: in case of irreversible damages or exhaustion of satellite cells proliferation potential, in fact, autologous muscle transplantation is needed, with all the related drawbacks. *In vitro* engineered structures could represent a valid alternative for tissue replacement, with the advantages of the elimination of donor site morbidity and reduction of operative time and rehabilitation times [1], [2]. Another recent important research field is represented by bio-hybrid systems, in particular bio-hybrid actuators. It has been actually argued that actuators based on living contractile cells cultured on properly engineered substrates could be used in future machines, thus overcoming many intrinsic limitations of current artificial actuators [3], [4], [5]. Finally, skeletal muscle tissue engineering has been recently proposed as a new approach to produce meat *in vitro* [6]. All these challenges require integrated efforts towards the development of 2D and 3D engineered scaffolds and a series of lateral technologies able to provide cells with the physical cues required for their orientation, fusion, differentiation and maturation.

Concerning cell alignment, it is well known that skeletal muscle cell orientation is strongly influenced by substrate micro- and nano-structuration [7]. In particular, it has been shown that an anisotropic topography is an effective means to induce myoblast alignment, due to the contact guidance theory, and that aligned ridges or grooves with widths ranging from 5 to 25 μm promote a high myoblast alignment and their fusion in well-aligned myotubes [8], [9], [10], [11], [12]. Sub-micron ridges/grooves or fibers have been demonstrated to promote a higher cell differentiation, but in general they do not further improve cell alignment [13], [14].

Substrate mechanical stiffness also deeply influences cell behaviour [15]. It has been demonstrated that skeletal muscle cells optimally differentiate by forming elongated and multinucleated myotubes when cultured on substrates characterized by tissue-like stiffness (10–15 kPa) [16], while much softer or much stiffer matrices hamper myotube formation or induce cell detachment.

Electrical stimulation has been demonstrated to have an important role in enhancing muscle cell differentiation, by providing electrical cues able to stimulate the expression of transcription factors governing differentiation along skeletal or cardiac lineages [17], [18]. Recently, an innovative means to provide cells with intracellular electrical stimuli has been proposed, based on piezoelectric nanoparticles stimulated by outer ultrasound sources [19]. Boron nitride nanotubes (BNNTs) are structural analogues of carbon nanotubes, in which alternating B and N atoms entirely substitute for C atoms in a graphitic like sheet. BNNTs possess many intriguing properties, including a strong piezoelectricity, which is the ability to generate electric energy when mechanically stressed [20]. Plain BNNTs in aqueous media form large clusters which hamper cellular up-take processes. To overcome this limitation, dispersion strategies of BNNTs have been proposed with a wide range of surfactants, such as glycol chitosan (GC), poly-L-lysine and polyethyleneimine, which wrap single nanoparticles with a polymeric coating, thus enabling their water-dispersion and facilitating cellular take-up. The biocompatibility of the polymer used also determines the cytocompatibility of polymer-coated BNNTs [21]. The safety of using BNNTs, alone or coupled with ultrasound stimulation, has been demonstrated in several studies *in vitro* [22], [23], [24] and, more recently, *in vivo* [25].

As mentioned, the piezoelectricity of BNNTs represents an intriguing feature, as such nanostructures (properly functionalized) can be used to convey physical stimuli within cells. Ultrasound-based stimulation of BNNTs internalized by cells showed impressive results with regard to neurite outgrowth in neuron-like PC12 cells [23] and was also proposed for C2C12 skeletal muscle cells [24], even if the effects of such stimulation on muscle differentiation were investigated only in the short run (up to 72 h).

Several efforts have been made in the last decades to find suitable matrices and physical stimulation means for skeletal muscle differentiation and functional maintenance. However, most groups have focused on only one or two of the numerous aspects affecting the development of muscle constructs, such as micro/nano-topography [8], [14], [26], [27], [28], [29], [30], matrix stiffness [16], [31], [32], [33], chemical cues [34], [35] and electrical stimulation [17], [36], [37], [38]. The combination of multiple mechanical, electrical, topographical and chemical cues constitutes an active field of research and it holds great promises for the commitment of stem cells towards specific cell phenotypes [39], but also for the late differentiation of skeletal muscle cells [40].

In this paper, we report the results obtained by combining a series of chemical, topographical, mechanical and electrical stimuli towards the realization of an engineered skeletal muscle-based system. We prepared polymeric free-standing thin substrates showing optimal mechanical features for skeletal muscle differentiation and we investigated the influence of micro-topography and BNNT-mediated stimulation on the differentiation of C2C12 skeletal myoblasts co-cultured with normal human dermal fibroblasts (nHDFs), also highlighting the possible role of fibroblasts in muscle differentiation (e.g., related to cytokine production). In order to facilitate the article reading, a list of the abbreviations used is also provided (Table S1).

## Materials and Methods

### Flat and Micro-grooved Polyacrylamide Gel Preparation and Functionalization

**Figure 1:**
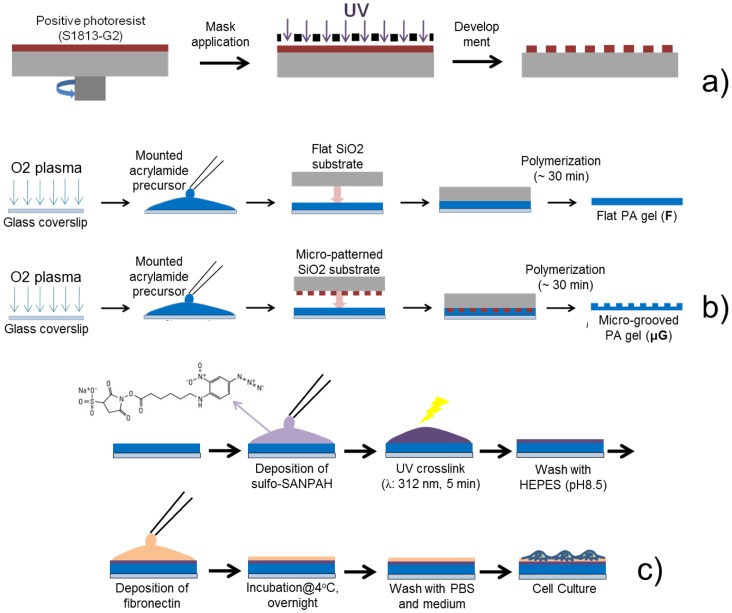
Schematics of the experimental procedure followed for fabricating and functionalizing polyacrylamide gels. (a) Fabrication of µG Si molds by means of photolithographic processes; (b) procedure to obtain free-standing flat and µG hydrogels; (c) hydrogel functionalization procedure, based on the activation of a photoresponsive cross-linker and fibronectin deposition. For simplicity, only a flat sample is reported.

The hydrogels used in this study were prepared by modifying existing protocols for polyacrylamide (PA) gel preparation [41], [42]. Figure 1 outlines the fabrication steps needed for the development of such thin, free-standing gels. Briefly, microfabricated Si molds (Figure 1a) and a pre-treatment of glass substrates with O_2_ plasma (Figure 1b) were used to obtain flat or micro-grooved free-standing thin hydrogels, which were then properly functionalized by cross-linking fibronectin on their surfaces (Figure 1c), thus allowing their use as substrates for cell culture.

In details, glass coverslips were treated with O_2_ plasma (200 mTorr pressure, 6.8 W, 60 sec, Plasma Cleaner, Gambetti s.r.l. Italia), then covered with 100 μL of a solution composed of 10% acrylamide/BIS-acrylamide (29∶1 ratio, Sigma-Aldrich) in dd-H_2_O supplemented with 1/200 volume of 10% ammonium persulfate (Sigma) and 1/2000 volume of N, N, N′, N′-tetramethylethylenediamine (TEMED, Sigma). Micro-grooved Si molds, obtained by means of standard photolithographic processes or flat Si molds, both previously treated for 20 min with trymethylchlorosilane (Carlo Erba), were placed upside-down on the top of the acrylamide solution drop. After ∼30 min, the polymerization process was completed; the Si molds were detached from the PA gels, which were rinsed with 50 mM 4-(2-hydroxyethyl)-1-piperazineethanesulfonic acid (HEPES, Sigma) at pH = 8.5 on a shaker. After a few hours in liquid, the hydrogels detached from the supporting glass coverslips, thus allowing free-standing substrates to be obtained.

To properly functionalize them, hydrogels were covered with 1 mM sulfosuccinimidyl 6-(4′-azido-2′-nitrophenylamino)hexanoate (sulfo-SANPAH, Thermo Scientific) and exposed for 5 min to a 30 W UV lamp at a distance of 15 cm. Darkened sulfo-SANPAH solution was removed and the photoactivation procedure was repeated. Gels were washed with two changes of 50 mM HEPES (pH = 8.5), 15 min each, on a shaker, and then treated with a 20 μg/mL fibronectin (Sigma) that was allowed to react overnight at 4°C. Coated gels were then washed with phosphate buffered saline (PBS, EuroClone) and soaked for ∼ 45 min in culture medium at 37°C before cell seeding.

### Si Mold and PA Gel Characterization

Microfabricated Si molds were characterized by scanning electron microscope (SEM) and atomic force microscope (AFM). SEM images were obtained with an EVO MA15 SEM (Zeiss) equipped with LaB_6_ source and working at a 10 kV accelerating voltage. AFM scans were performed by using an Innova Scanning Probe Microscope (Veeco). Measurements were performed in air, at room temperature and operating in tapping mode, with oxide-sharpened silicon probes (RTESPA-CP) at a resonant frequency of ∼ 300 kHz. AFM images were processed by means of a Gwyddion SPM software analysis tool. Bright field images of PA gels showing the micro-grooves transferred from molds to gels were obtained by an optical microscope (Hirox KH 7700 Digital 3D video microscope with objective lenses covering a magnification range from 35X to 7000X). PA gel thickness was assessed by means of a profilometer (Kla-Tencor P6, low-force head configuration surface profiler with 2 μm, 60 degree stylus radius). Gel mechanical properties were evaluated by performing traction tests with an INSTRON 4464 Mechanical Testing System, using a ±10 N load cell. Gels were carefully taken with tweezers and placed between two *ad hoc* designed aluminium clamps. All samples were pulled at a constant speed of 5 mm/min, until reaching sample failure. Data were recorded at a frequency of 100 Hz; stress was calculated as the load divided by the cross-section area of tensile specimens, while strain was calculated as the ratio between the extension and the initial length of tensile specimens. The elastic modulus for each tested sample was then calculated starting from its stress/strain curve. Fibronectin coating quantitative characterization was performed by leaving fibronectin-coated hydrogels in PBS and analyzing the sample supernatant for 14 days after functionalization. At this aim, we analyzed the protein (fibronectin) content in the supernatant by using a Bio Tex SynergyMX spectrophotometer reading absorbance at 280 nm. A 2 μl drop of supernatant was placed in each well of a TAKE 3 plate and the data were elaborated using Gen5 software. TRITC-fibronectin (20 μg/mL, Invitrogen) was then used to visually assess protein coating stability on sample surfaces. Samples were imaged at different time points to check if the protein (showing red fluorescence) remained on the gels after incubation in the cell culture medium. All fluorescence images were acquired by using an inverted fluorescence microscope (Eclipse Ti) equipped with TRITC, FITC and DAPI filters (Nikon), with a cooled CCD camera (DS-5MC USB2, Nikon) and with NIS Elements imaging software.

### Cell Cultures

Normal human dermal fibroblasts (nHDFs) were purchased from Lonza (Cat. # CC-2511). C2C12 myoblasts were purchased from ATTC (Cat. # CRL-1772). Both cell types were expanded in proliferation medium, composed of 90% Dulbecco’s Modified Eagle’s Medium (DMEM, Euroclone) supplemented with 10% Fetal Bovine Serum (FBS, Euroclone), 100 IU/mL penicillin (EuroClone), 100 μg/mL streptomycin (EuroClone) and 2 mM L-glutamine (Sigma). During culture, the cells were maintained at 37°C in a saturated humidity atmosphere containing 95% air and 5% CO_2_. nHDFs (passage <15) were seeded on the fibronectin-coated PA gels at a density of 5,000 cells/cm^2^. Twenty-four hours after seeding, they were treated to inhibit their mitotic activity and were provided with proliferation medium supplemented with 8 μg/mL mitomycin C (Sigma-Aldrich) for 2 h at 37°C. C2C12 cells (passage <5) were seeded on the top of the fibroblast layer at a density of 60,000 cells/cm^2^, thus assuring complete cell confluence 24 h after seeding. Twenty-four hours after C2C12 cell seeding, the proliferation culture medium was replaced with differentiation medium, composed of 98% DMEM, 1% FBS, 1% insulin-transferrin-sodium selenite (ITS, Sigma-Aldrich), 100 IU/mL penicillin, 100 μg/mL streptomycin and 2 mM L-glutamine. The differentiation medium was replaced daily and, starting from the third day of differentiation, it was also supplemented with 5 μg/mL AraC (Pfizer), to contrast persistent C2C12 proliferation at early stages of differentiation.

### Image Analysis

Both bright field and fluorescence images were analyzed using ImageJ, a free software available at http://rsbweb.nih.gov/ij/download.html. Cell alignment was evaluated by measuring the angle formed by the cell’s major axis and a properly chosen reference axis. The minimum angle value of 0° corresponded to a perfect alignment of cells along the chosen axis, whereas a value of 45° corresponded to a perfectly random cell orientation on the substrate. For flat samples, an arbitrary axis was chosen, while for micro-grooved samples the groove axis was chosen as the reference axis. The fusion index is a useful parameter that permits to quantify the differentiation level of *in vitro* cultured myotubes on the basis of the presence of multinucleated structures [31]. The fusion index was determined by dividing, for each fluorescence image, the total number of nuclei in myotubes (≥2 nuclei) by the total number of counted nuclei.

### BNNT Preparation, Characterization and Stimulation

BNNTs (purchased from the Nano and Ceramic Materials Research Center, Wuhan Institute of Technology, Wuhan, Hubei, China) were produced by using an annealing method from boron containing precursors. Details of the sample, provided by the supplier, include boron nitride >98.5 wt%, yield >80%. If not differently specified, chemicals were purchased from Sigma-Aldrich (St. Louis, MO, USA). Glycol chitosan (GC) was used for the dispersion and stabilization of BNNTs. Dispersion was prepared with PBS. BNNTs (10 mg) were mixed with 10 ml of a 0.1% GC solution in a polystyrene tube. The samples were sonicated for 12 h (by a Bransonic sonicator 2510, Danbury, CT, USA) with an output power of 20 W for all the experiments, resulting in a stable GC-BNNT dispersion by the non-covalent coating of the nanotube walls with GC. Microphotographs of the final dispersion of BNNTs were obtained with a FEI 200 FIB microscope. BNNTs were added to the differentiation medium of BNNT-treated samples at a concentration of 10 μg/mL, thus inducing their internalization by cells. Internalized BNNTs were stimulated daily, during cell differentiation, by means of outer ultrasound sources. Stimulation was carried out by using a Bransonic sonicator 2510 and by partially immersing the culture plate (properly sailed with parafilm) in the ultrasound bath for 10 s every day, applying 20 W power and 40 kHz frequency. The choice of this stimulation protocol derived from preliminary tests on co-cultured cells, which highlighted that an higher stimulation frequency (more than one stimulation per day) and/or a greater time of stimulation (>10 s) implied an increased cell mortality, especially at later stages of differentiation.

### Inductive Coupled Plasma Mass Spectrometry (ICP-MS) Analysis

BNNT internalization was assessed by measuring the boron content in cell lysates. Cells were extensively rinsed with PBS to avoid any residual of extracellular BNNTs, then they were trypsinized and collected in a centrifuge tube. Cells were then provided with 1 mL of nitric acid (68% in H_2_O) and incubated overnight at room temperature, in order to completely disrupt any organic component. Afterwards, the samples were freeze-dried and the presence of boron in the samples was evaluated by ICP-MS (Thermo X Series). To this purpose, samples were digested overnight in the presence of hydrofluoric acid (0.1 mL, 40%, (w/v)) and ultrasounds. Then, 9.9 mL of aqueous nitric acid solution (2% (w/v)) were added. The samples (n = 3) were analyzed by ICP-MS for the quantification of internalized boron. Boron content (directly proportional to BNNTs internalized by cells) was thus quantitatively assessed.

### Transmission Electron Microscopy (TEM) Imaging and Electron Energy Loss Spectroscopy (EELS) Analysis

Scanning TEM-high angle annular dark field (STEM-HAADF) was employed to visualize C2C12 myoblasts co-cultured with nHDFs, incubated with GC-conjugated BNNTs. Samples were fixed in 2% glutaraldehyde in 0.1 M cacodylate buffer for 2 h, washed several times in the same buffer, post-fixed in 1% osmium tetroxide in d-H_2_O, stained overnight at 4°C in 0.5% uranyl acetate in d-H_2_O, dehydrated in a graded ethanol series, and embedded in SPURR resin. To release the embedded cells from their substrates, the samples were transferred between liquid nitrogen and hot water. Planar and transverse sections of about 70 nm in thickness were cut with a diamond knife on an ultramicrotome Leica EM UC6. STEM-HAADF investigations were carried out using a Jeol JEM-2200FS TEM, equipped with a field emission gun operated at 200 kV and with an Omega filter. To identify the BNNTs, electron energy loss spectra (EELS) were acquired by scanning a 1 nm electron beam on selected features in STEM-HAADF mode.

### In Vitro Assays

With regard to genetic analyses, the expression of ten genes responsible for skeletal muscle differentiation was evaluated at two time points (D3 and D7) by quantitative real-time polymerase chain reaction (qRT-PCR). Total RNA from experimental groups was isolated using a protocol with TRIzol (Invitrogen) and Rneasy Minikit (Qiagen). After RNA extraction, cDNA was prepared from 1 μg RNA using Taqman Reverse transcription reagents (AppliedBiosystems). The reference sample was represented by C2C12 cells in proliferation state, at 70% confluence on polystyrene flasks, not provided with any differentiative stimulus. qRT-PCR was performed using Power SYBR Green PCR Master Mix and detection was carried out by means of an ABI PRISM 7500 System (Applied Biosystems). Quantification of target genes was performed in respect of the reference GAPDH gene, using the following formula: relative expression = 2^[–(Ct sample – Ct GAPDH)]^. The mean minimal cycle threshold values (Ct) were calculated from quadruplicate reactions. Then, the relative gene expression in each experimental group was normalized to the relative gene expression found in the reference sample. Regarding immunocytochemistry procedures, cells were fixed at the timepoints (D3 and D7) by using 4% paraformaldehyde (Sigma-Aldrich) in PBS for 15 min and permeabilized by using 0.1% Triton X-100 in PBS for 15 min, following a standard procedure. Oregon Green® 488 phalloidin (Invitrogen) and 1 µM DAPI (Invitrogen) were used to stain F-actin and cell nuclei, respectively, at D3. Fluorescence images were acquired by a confocal fluorescence microscope (LSM 510 Meta, Carl Zeiss), equipped with TRITC, FITC and DAPI filters. At D7, cells were stained for α-actinin (anti-α-actinin, from abcam) and myosin heavy chain (MHC) (anti-MHC, from Santa Cruz Biotechnology) with respectively Oregon green- and rhodamine-conjugated IgGs (Invitrogen), used as secondary antibodies. Fluorescence images were acquired by confocal microscope.

### Cytokine Measurements

Fibroblast (nHDF) culture supernatants were assayed for cytokines using a Bio-plex human 17-plex panel immunoassay kit (Bio-Rad, http://www.bio-rad.com) and cytokine concentrations were determined using Bio-Plex Manager 5, according to manufacturer’s instructions. The 17-Plex panel consisted of the following analytes: interleukin-1 (IL-1β), IL-2, IL-4, IL-5, IL-6, IL-7, IL-8; IL-10, IL-12(p70), IL-13, IL-17, granulocyte colony-stimulating factor (G-CSF), granulocyte/macrophage colony-stimulating factor (GM-CSF), interferon (IFN-γ), monocyte chemotactic protein (MCP-1 (MCAF)), macrophage inﬂammatory protein (MIP-1β), and tumor necrosis factor (TNF-α). Supernatant media samples were collected, centrifuged and frozen. Samples and controls were run in triplicate, standards and blanks in duplicate. Only seven analytes were detected; the others were below the lower detection limit.

### Calcium Transients Imaging

C2C12 cultures at D7 were loaded with 5 µM Fura-2 pentacetoxymethyl ester (Sigma-Aldrich) in Krebs’-Ringer’s-HEPES solution (KRH, with the following composition (in mM): 125 NaCl, 5 KCl,1.2 MgSO_4_, 1.2 KH_2_PO_4_, 2 CaCl_2_, 6 glucose, and 25 HEPES-NaOH, pH 7.4) containing 10 mg/ml bovine serum albumin (Sigma-Aldrich) for 45 minutes at 37°C, washed in the same solution and transferred to the recording chamber of an inverted microscope (Leica DMI6000) equipped with a calcium imaging unit Polychrome V (TILL Photonics, Germany). Data were collected with Imaging Worckbench 6.0 software. Regions of interest (ROI), corresponding to multinucleated myotubes, were properly drawn. After a short period for baseline acquisition, C2C12 cells were sequentially stimulated with 2 mM caffeine (Sigma-Aldrich) and 100 µM Acetylcholine (Sigma-Aldrich), to evaluate the functional response of myotubes grown in the different experimental conditions.

### Statistical Analyses

The data collected were subjected to analysis of variance in order to evaluate the statistically significant differences among samples. A *t*-test was performed for comparison between two groups, while Holm–Sidak tests were performed for comparisons among several groups. Significance was set at 5%.

## Results and Discussion

### Characterization of Polyacrylamide Substrates

**Figure 2:**
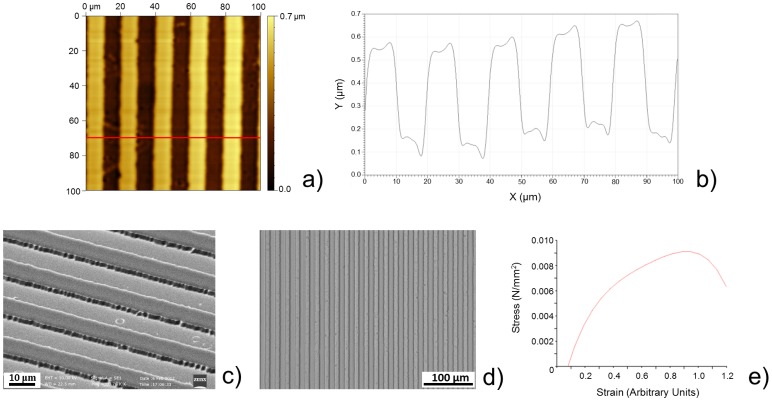
Results of topographical and mechanical characterization. AFM image (100 µm × 100 µm) of a micro-grooved (µG) Si mold (a) and height profile (b) corresponding to the red line in the AFM image; (c) SEM image of a μG Si mold; (d) optical image showing the micropattern efficiently transferred from Si mold to PA gel surface; (e) representative stress-strain curve for PA gels. Mechanical properties were evaluated by testing at least 20 samples for each gel type (flat and μG).

Atomic force microscope (AFM) characterization and scanning electron microscope (SEM) imaging of the microfabricated molds revealed the presence of uniformly distributed 10 μm-wide, 10 μm-spaced and ∼400 nm-thick micro-grooves on the silicon (Si) surface (Figure 2a–c). These sizes have already proved to be effective in aligning skeletal muscle cells [10], thus making them suitable for our purpose (i.e., to create a strongly anisotropic muscle construct). By keeping the microfabricated molds upside-down on a PA solution during the polymerization process (Figure 1b), we succeeded in efficiently transferring micro-topography from the mold to the gel (Figure 2d).

After PA polymerization and mold removal, the hydrogels were placed in a liquid solution to enable substrate detachment from the glass coverslips. Free-standing PA gels were then collected on supporting Si substrates and their thickness was measured by using a profilometer. Gel mechanical properties were also assessed by means of traction tests (Figure 2e). The results, summarized in Table 1, revealed that both flat (F) and micro-grooved (μG) substrates had a thickness of 10–15 μm and an elastic modulus of ∼ 14 kPa. Low substrate thickness is crucial for envisioning PA gels as parts of a contractile bio-hybrid actuator able to compliantly favour cell contraction [4]; instead, the matrix elastic modulus, as previously described, strongly affects the differentiation ability of skeletal myoblasts [16]. The PA gels described in this study, therefore, match all the required features, in terms of topography, thickness and stiffness.

**Table 1:** Thickness, elastic modulus and surface topography of F and μG PA gels. Mean values and standard deviations are reported.

*Substrate*	*Thickness*	*Young’s modulus*	*Topographical cues*
Flat PA gels	13.1±3.7 µm	14.2±1.8 kPa	–
µG PA gels	12.2±5.2 µm	14.7±1.5 kPa	10 µm-wide grooves

**Figure 3:**
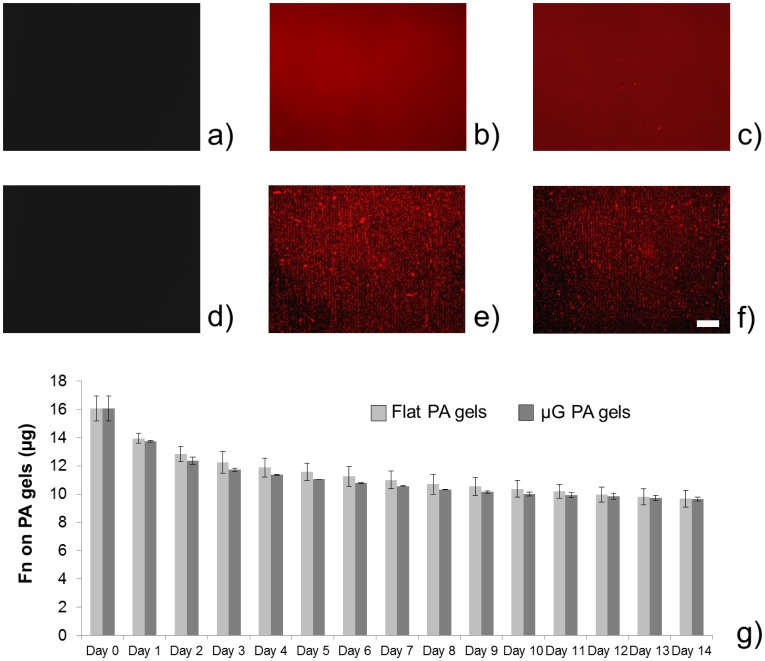
Stability of fibronectin coating on the polyacrilamide gels. Gels were treated with sulfo-SANPAH (see Experimental section), coated with a 20 μg/ml fibronectin solution, incubated overnight at 4°C and maintained in culture medium (a-f) or PBS (g) for 2 weeks. Control images of F (a) and μG (d) PA gels show that no autofluorescence can be detected in the red channel; TRITC-fibronectin is clearly visible both on F (b) and μG (e) gels 24 h after treatment, and the coating is maintained on both F (c) and μG (f) gels 2 weeks after treatment, replacing culture medium every day. Quantitative data (reporting the difference between the initial amount of protein placed on the gel and the protein released in the supernatant, daily measured by means of absorbance readings) show that few amounts of fibronectin detached from the gels during the observation period, thus confirming that the protein coating is stable over time (g). Daily absorbance readings were performed on three independent samples for each sample type.

The cross-linking of an extracellular matrix (ECM) protein, such as fibronectin, on PA gel surfaces is of primary importance for the stable anchoring of cells to the polymeric sheet. PA is actually a cell-repulsive material; a functional coating, therefore, is essential for cell culture maintenance [43]. The long-term stability of such coating avoids cell detachment after a few days of culture, thus allowing the development and maintenance of skeletal muscle constructs. We were able to maintain a stable fibronectin coating on both F and μG PA gels for at least 14 days, as shown in Figure 3.

### nHDF and C2C12 Alignment and BNNT Internalization

We used a co-culture of nHDFs (normal human dermal fibroblasts) and C2C12 (mouse myoblasts) cells since, as known from previous literature, the presence of fibroblasts co-cultured with skeletal muscle cells enhances myotube formation and maturation [44].

**Figure 4:**
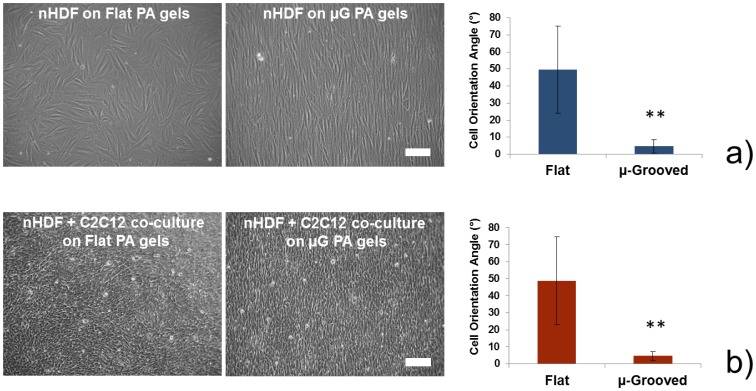
Cell orientation on F and μG hydrogels 24 h after seeding. (a) Bright field images (scale bar = 100 μm) and quantitative cell orientation angle measurements reveal that nHDFs are isotropically oriented on flat PA gels (orientation angle close to 45°, corresponding to total isotropy), while they show a strong anisotropic orientation on μG PA gels (orientation angle close to 0°, corresponding to total anisotropy), with a clear alignment along the micro-groove axis; (b) bright field images (scale bar = 100 μm) and quantitative cell orientation angle measurements reveal that C2C12 cells, cultured on the top of the fibroblast layer, are also randomly oriented on flat PA gels and strongly aligned along the micro-groove axis on μG PA gels. For quantitative analyses, five low-magnification images were elaborated for each sample type and for each image at least 100 cells were analyzed. ** = p<0.01.

Fibroblast orientation on fibronectin-coated PA gels was evaluated 24 h after cell seeding. Figure 4a shows bright field images of nHDFs cultured on F and μG gels. An isotropic cell distribution was clearly visible on F samples, while a strong preferential alignment of cells along the groove axis was found on μG samples. Quantitative analyses of cell orientation angles confirmed these qualitative evidences. C2C12 cells were cultured on the top of the fibroblast layer and their orientation was also evaluated 24 h after seeding (Figure 4b). Interestingly, C2C12 cells also kept a random orientation in the case of F substrates and a strong anisotropic orientation (with cell bodies aligned along the groove axis) in the case of μG substrates. Quantitative data confirmed the evidences provided by bright field images. Therefore, we were able to align the co-culture along a preferential direction, which was a pre-requisite to obtain an efficient skeletal muscle differentiation.

**Figure 5:**
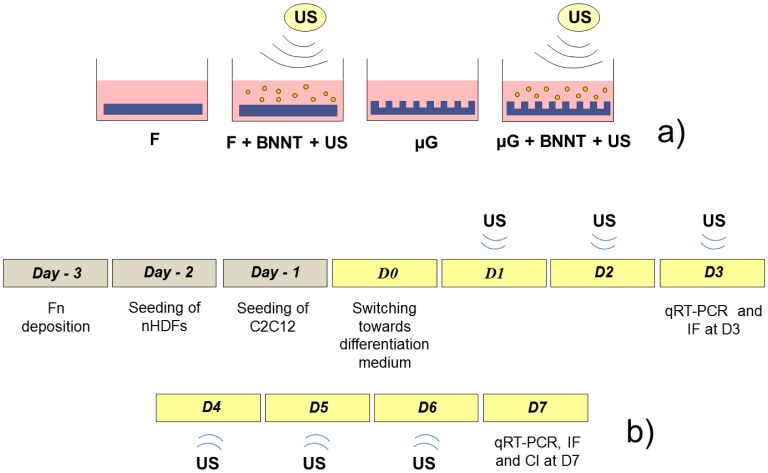
Schematics of the experimental layout. The different sample types are schematically represented in (a). Cells cultured on flat PA gels are provided with simple differentiation medium (F) or with differentiation medium supplemented with 10 μg/mL BNNTs and stimulated by outer ultrasound sources (F+BNNT+US). Similarly, cells cultured on micro-grooved PA gels are provided with simple differentiation medium (μG) or with differentiation medium supplemented with 10 μg/mL BNNTs and stimulated by outer ultrasound sources (μG+BNNT+US); (b) experiment timeline: D0 is set as the time point for the beginning of the differentiation process; from D1 to D6 BNNT-treated samples were provided with a daily ultrasound stimulation (5 s each). D3 and D7 are set as intermediate and final time points, respectively.

Four sample types were considered in the study, namely: cells cultured on flat and micro-grooved PA gels and cells cultured on the same substrates, but also provided with 10 μg/ml BNNTs in the culture medium and daily stimulated with ultrasounds (Figure 5a).

Previous literature highlighted that providing different cell types (including C2C12 cells) with culture medium supplemented with 10 μg/ml BNNTs induced nanoparticle internalization without significant cytotoxic effects [23], [24]. It has already been demonstrated that non-combined BNNT internalization or ultrasound (US) stimulation have no significant effects on cell behaviour [21], [22], [23]. The experiment layout (depicted in Figure 5b) included a daily US stimulation (for the BNNT+US treated samples) during muscle cell differentiation.

**Figure 6:**
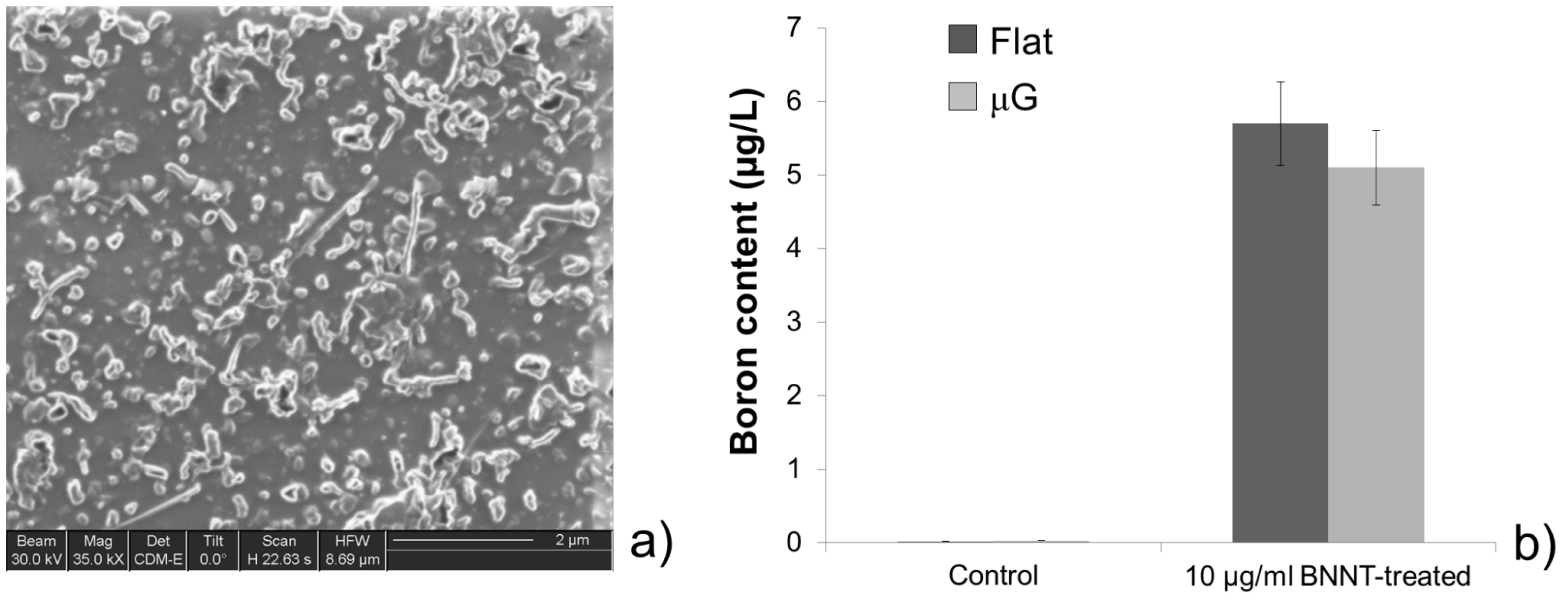
Characterization of CG-BNNT dispersion and results of quantitative internalization tests. (a) FIB image of a CG-BNNT (10 μg/ml) dispersion; (b) elemental analysis performed by ICP-MS analysis, revealing boron content in BNNT-treated cell lysates (on both flat and μG PA gels) and in controls (non-BNNT-treated cells on both hydrogel types). Analyses were performed on cells cultured on three gels for each sample type.

We prepared BNNT conjugated with GC (Figure 6a) and we dispersed them at a concentration of 10 μg/ml in the cell culture medium of BNNT-treated samples (we also supplemented the cell culture medium of non-BNNT-treated samples with only 10 μg/ml GC, as control). Twenty-four hours after providing cells with GC-containing or BNNT-containing culture medium, we evaluated nanoparticle internalization by ICP-MS analysis (Figure 6b). Results showed an appreciable internalization of nanoparticles by treated cells, while no boron traces were found in the control group.

**Figure 7:**
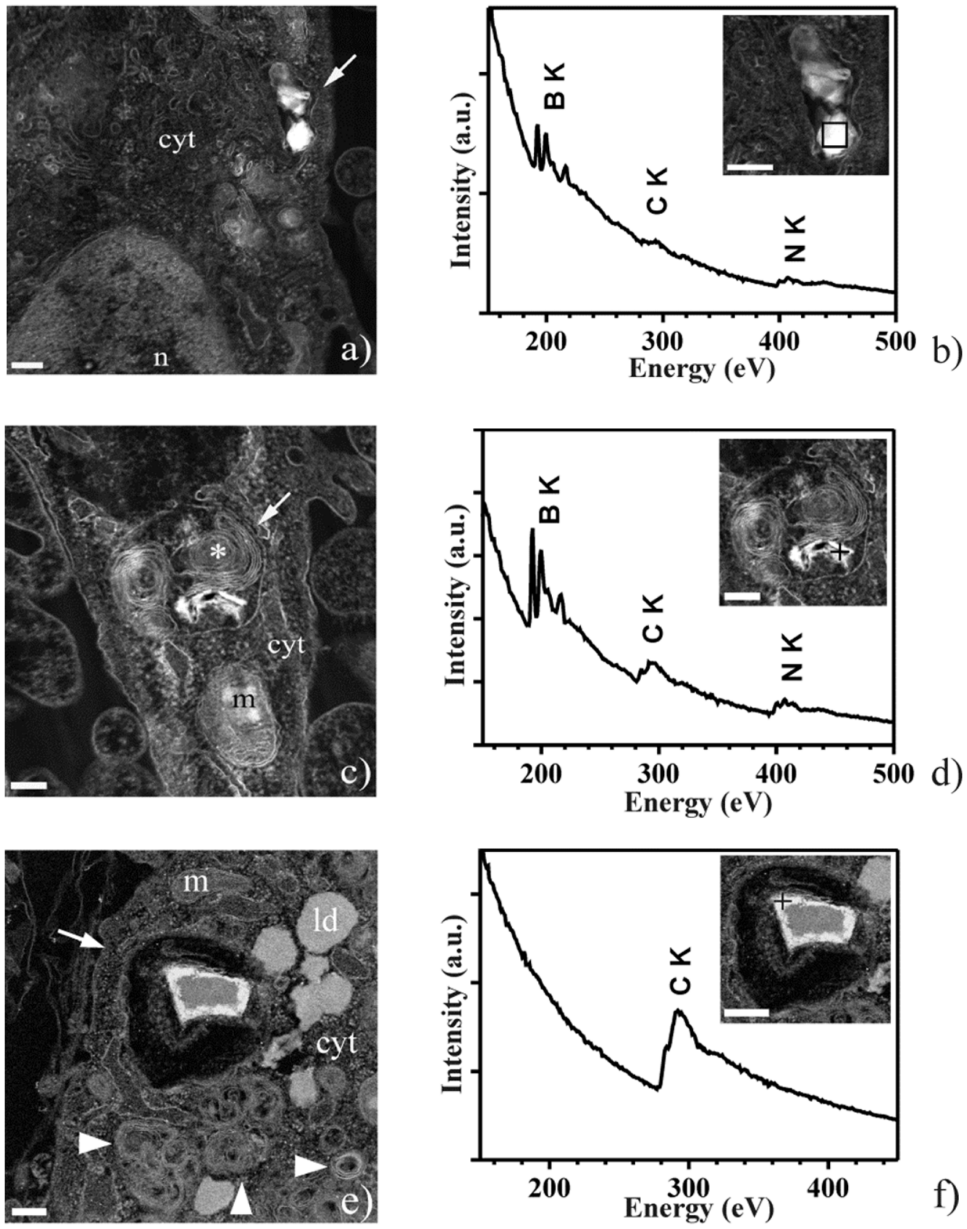
Proof of presence and intracellular localization of GC-conjugated BNNTs inside C2C12 myoblasts co-cultured with nHDFs via scanning TEM-high angle annular dark field (STEM-HAADF) coupled with energy electron loss spectroscopy (EELS). (a) STEM-HAADF image of part of a myoblast. The arrow points to an early endosome containing the GC-conjugated BNNTs. (b) EEL spectrum collected from the area boxed in the inset, showing the core-loss K edges of B and N, confirming that the selected nano-object is indeed a bundle of BNNTs. (c) STEM-HAADF image of part of another myoblast. The arrow points to a late endosome containing the GC-conjugated BNNTs. Note the multilamellar stack (asterisk) that is inside the late endosome. (d) EEL spectrum collected from the area pointed in the inset (+), confirming the presence of BNNTs. (e) STEM-HAADF image of a portion of a fibroblast showing several multilamellar bodies (arrowheads) close to an endosome (arrow). (f) EEL spectrum collected from the feature pointed in the inset (+), showing that the selected object does not contain BNNTs. Abbreviations: cyt, cytoplasm; ld, lipid droplet; m, mitochondrion; n, nucleus. Scale bars are 200 nm in panels (a-d), 300 nm in panel (e) and 400 nm in (f).

In agreement with ICP-MS results, STEM-EELS analysis performed on sections of C2C12 myoblasts co-cultured with nHDFs showed the presence of GC-conjugated BNNTs not only in early endosomes (Figure 7a,b), but also in their maturative forms, the late endosomes, characterized by internal residual membranes arranged in multilamellar stacks (Figure 7c,d). No free nanostructures were observed inside the cell cytoplasm, thus suggesting their internalization through endocytosis. Inside both endosomal compartments the nanostructures appeared as electron-dense clusters, irregular in shape, which the EELS mapping clearly revealed to consist of both boron (B) and nitrogen (N), as expected (Figure 7a–d). Intriguingly, we did not observe the presence of boron nitride nanotubes in any of the cell analyzed belonging to the fibroblast layer underneath the C2C12 cells (Figure 7e,f).

### Effects of Topographical and Intracellular Electrical Stimuli on Skeletal Muscle Differentiation

**Figure 8:**
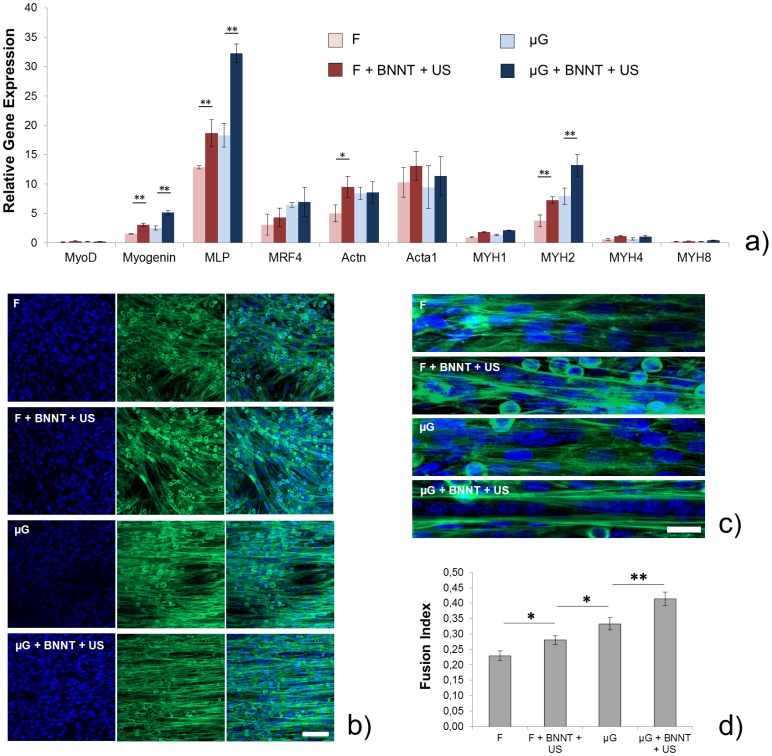
Evaluation of skeletal muscle differentiation for the different samples at D3. (a) Relative gene expression levels for ten genes important for skeletal muscle differentiation, compared between the different experimental groups (F, F+BNNT+US, μG and μG+BNNT+US). mRNA analyses were run in quadruplicate; low magnification (b, scale bar = 100 μm) and high-magnification (c, scale bar = 25 μm) confocal fluorescence images of randomly oriented or aligned myotubes on the different samples. F-actin is stained in green, nuclei are stained in blue; (d) fusion index for C2C12 cells cultured on the different sample types, determined by dividing the total number of nuclei in myotubes (>2 nuclei) by the total number of nuclei counted in the image. Five low-magnification images were elaborated for each sample type and at least 25 myotubes were analyzed for each image. * = p<0.05, ** = p<0.01.

Cell co-cultures were trypsinized or fixed in paraformaldehyde and then permeabilized for gene profiling or protein immunostaining, respectively, at two time points, namely day 3 (D3) and day 7 (D7). At D3, we performed qRT-PCR analyses on ten genes relevant for skeletal muscle differentiation in mouse cells (Table S2), such as MyoD, Myogenin, Muscle LIM Protein (MLP), MRF4, α-actinin (Actn), sarcomeric actin (Acta1), myosin heavy chain (MHC)-IId-x (MYH1), MHC-IIa (MYH2), MHC-IIb (MYH4) and perinatal MHC (MYH8) [45]. We found important differences in gene expression levels among the tested samples (Figure 8a). Micro-topography and BNNT-mediated stimulation both enhanced Myogenin, MLP and MYH2 expression, with a clear synergic effect in μG+BNNT+US samples. Actn expression was also markedly different between F and F+BNNT+US samples, whilst no significant differences were found in terms of Actn expression between μG and μG+BNNT+US samples. Myogenin and MLP have a key role in the early stage of myogenesis; their marked overexpression in F+BNNT+US samples in comparison with F samples, as well as in µG+BNNT+US in comparison with µG samples, suggests that the intracellular electrical stimulation, mediated by the internalized BNNTs localized in the endosomes of muscle cells, has a dramatically positive effect on the myogenic process. A relatively late differentiation marker, such as MYH2, is also overexpressed in the samples stimulated by the combination BNNT+US. This suggests that the differentiation process is highly accelerated on these samples.

At the same time point (D3), we also performed F-actin and nuclei staining on cells cultured in the different experimental conditions, in order to visualize their cytoskeletal organization. Fluorescence images revealed that micro-topography clearly induced myotube alignment along the groove axis and that μG+BNNT+US samples were characterized by a higher percentage of elongated multinucleated myotubes, compared to the other sample types (Figure 8b, c). Low magnification images were then analyzed, in order to quantitatively evaluate the differentiation grade of cells on the different samples. The fusion index was determined by dividing the total number of nuclei in myotubes (>2 nuclei) by the total number of nuclei counted in the image [31]. Results showed that the cell fusion index progressively and significantly increased from F samples to F+BNNT+US, μG and μG+BNNT+US samples (Figure 8d).

In order to investigate the possible role of fibroblasts in the marked increase in the differentiation ability of C2C12 cells on μG+BNNT+US samples, we analyzed both ECM-related gene expression (Table S3) and fibroblast cytokine production at D3. It is actually known that the development of *in vitro* bioengineered skeletal muscle is profoundly influenced by cell-matrix interactions: an optimized matrix composition can produce a significant cell hypertrophy and a prolonged Ca^2+^ transient half-width [46]. Parallely, specific cytokines produced by fibroblasts have shown their potential as promoters of myotube development in chimeric co-cultures [44].

**Figure 9:**
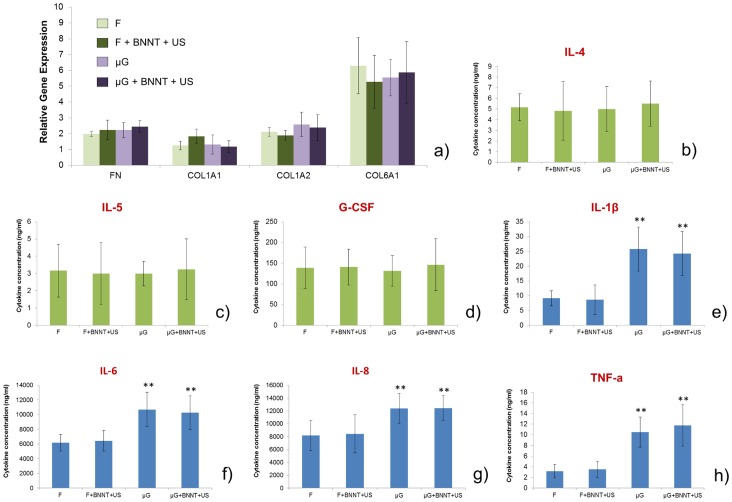
Investigation of the role of human fibroblasts in the skeletal muscle differentiation process. (a) Relative gene expression levels for genes encoding the production of ECM proteins for nHDFs cultured on the different sample types at D3. The investigated genes encoded the expression of fibronectin (FN), collagen type 1, alpha 1 (COL1A1), collagen type 1, alpha 2 (COL1A2) and collagen type 6, alpha 1 (COL6A1). No significant differences were found between the different sample types; (b-h) cytokine concentration measured in the surnatant at D3. The levels of interleukin 4 (IL-4), interleukin 5 (IL-5) and granulocyte colony stimulating factor (G-CSF) were not affected by topography, nor by BNNT-mediated stimulation, while the levels of interleukin 1b (IL-1b), interleukin 6 (IL-6), interleukin 8 (IL-8) and tumor necrosis factor-alpha (TNF-A) were affected by topography, but not by BNNT-mediated stimulation. Samples and control were run in triplicate. ** = p<0.01.

Results showed that no differences can be found in the expression levels of genes encoding ECM proteins such as fibronectin and different types of collagen for the different sample types (Figure 9a), suggesting that topographical and ultrasound-mediated stimuli do not influence fibroblast ECM protein production. With regard to cytokine production, we found that the amount of interleukin 4 (IL-4), interleukin 5 (IL-5) and granulocyte colony stimulating factor (G-CSF) in the supernatant was not affected by topography, nor by BNNT-mediated stimulation (Figure 9b–d), while the production of interleukin 1β (IL-1β), interleukin 6 (IL-6), interleukin 8 (IL-8) and tumor necrosis factor-alpha (TNF-α) was affected by topography, but not by ultrasound-mediated stimuli (Figure 9e–h).

As evidenced in Figure 7, BNNTs are not internalized by fibroblasts in our co-culture system. This may explain the lack of differences between the stimulated and the non-stimulated samples. In both F+BNNT+US and µG+BNNT+US samples, in fact, no electrical stimuli are conveyed at intracellular level, due to the absence of the piezoelectric nanoparticles. As a consequence, ultrasounds only have a mechanical effect on cells, without significant effects on ECM protein and cytokine production. Topography has a significant effect only on four cytokines. The upregulation of IL-6 expression is known to promote myogenic differentiation of mouse skeletal muscle cells [47]. Despite IL-8 is usually associated with inflammation, it has been recently demonstrated that it limits the inflammatory response in muscle cells [48]. TNF-α and IL-1β are inflammatory cytokines, usually associated with the inhibition of myogenic differentiation [49], [50]. However, it has been recently evidenced that TNF-α is also a key activator of p38 MAPK, thus indicating its essential role in myogenesis [51].

In conclusion, the fibroblasts monolayer can function as an elastic support for the contracting myotubes (as shown in the following, contraction is enhanced by the physical stimuli provided) and perhaps it also provides protection of the sarcolemmal membrane from contraction-induced damage [44]. Also the components secreted by the fibroblasts (extracellular matrix components and growth factors) may contribute to the improved survival and differentiation of myotubes. The micro-grooved topography induces an upregulation of cytokines which contribute to both enhance (in the case of IL-6, IL-8 and TNF-α) and inhibit (in the case of IL-1β) myogenesis, but it represents anyhow a key pre-requisite to obtain highly aligned functional myotubes.

**Figure 10:**
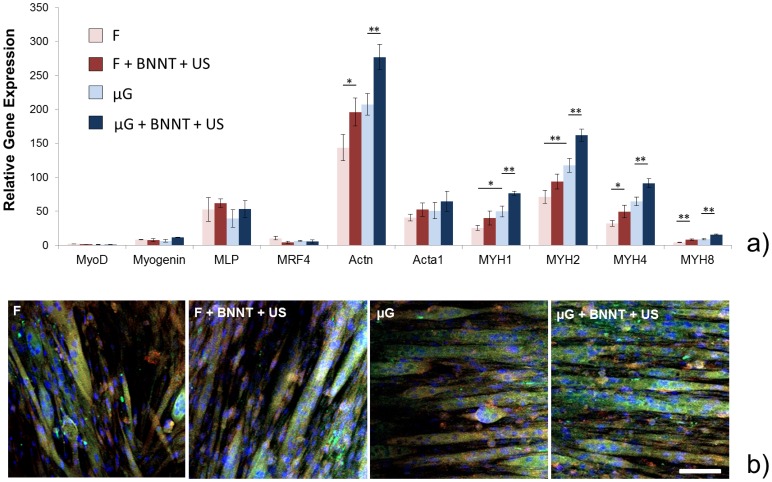
Evaluation of skeletal muscle differentiation for the different samples at D7. (a) Relative gene expression levels for ten genes important for skeletal muscle differentiation, compared between the different experimental groups (F, F+BNNT+US, μG and μG+BNNT+US). mRNA analyses were run in quadruplicate; (b) confocal fluorescence images of myotubes on the different samples: myosin heavy chain is shown in red, α-actinin is shown in green, nuclei are shown in blue. Scale bar = 100 μm. * = p<0.05, ** = p<0.01.

At D7, qRT-PCR analyses revealed that samples treated with BNNTs and ultrasounds were characterized by higher levels of Actn, MYH1, MYH2, MYH4 and MYH8 expression (Figure 10a).

Early differentiation-related genes were almost turned off in all the samples, confirming that cells were in a quite advanced differentiation stage. Thus, BNNT-mediated stimulation (and even more the synergy between BNNT-mediated stimulation and anisotropic micro-topography) revealed its ability to deeply affect the expression of genes encoding key proteins in the development of muscle contractile machinery, such as α-actinin and the different myosin isoforms.

At the same time point (D7), we performed immunostaining on cells cultured on the different sample types, thus obtaining fluorescence images of nuclei, myosin heavy chain and α-actinin (Figure 10b). The images confirmed that cells cultured on μG+BNNT+US samples form longer and wider multinucleated myotubes compared to the other samples. The presence of peripheral nuclei in the myotubes is an index of an advanced differentiation stage [44]. It can be noticed that the myotubes on µG and µG+BNNT+US samples show an higher number of peripheral nuclei in comparison with those on F and F+BNNT+US counterparts.

**Figure 11:**
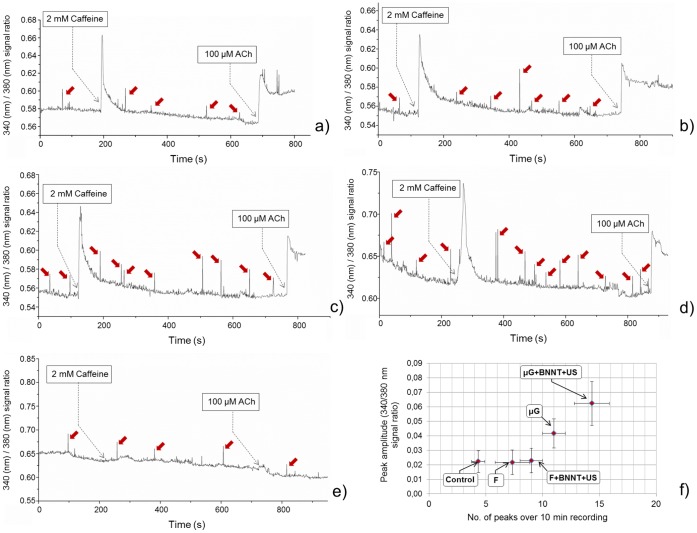
Intracellular (340 nm)/extracellular (380 nm) [Ca^2+^] ratio signals recorded in ROI corresponding to single myotubes. The different experimental conditions were tested: F (a), F+BNNT+US (b), μG (c) and μG+BNNT+US (d). As control, the signal corresponding to a single undifferentiated C2C12 cell was also acquired (e). Red arrows show peaks of spontaneous intracellular [Ca^2+^] entry, dashed arrows indicate the time points corresponding to the addition of 2 mM caffeine and 100 μM ACh in the chamber. (f) Quantitative analysis of calcium-related spontaneous peaks, in terms of total number and amplitude. At least 5 (10 min-long) signal tracks and a minimum of 25 peaks were analyzed for each sample type.

In order to get an insight on myotube functionality, we performed calcium imaging experiments at D7, by selecting multinucleated structures as regions of interest (ROI) for the different sample types and recording the intracellular/extracellular [Ca^2+^] ratio signal in basal conditions and after chemical stimulation, obtained by means of caffeine and acetylcholine (ACh) solutions, respectively. Figure 11 shows the signals acquired for the different samples, over about 10 minutes of [Ca^2+^] flux recording. μG+BNNT+US samples (Figure 11d) were characterized by more frequent and higher peaks of spontaneous intracellular [Ca^2+^] entry compared to the other sample types, as also highlighted by the quantitative analysis of calcium peaks (Figure 11f). This is known to be related to membrane oscillatory mechanisms, typical of a more advanced myogenic differentiation state [52]. More in details, concerning the number of spontaneous peaks, F samples showed a slightly but significantly (p = 0.048) higher value in comparison with undifferentiated control cells. No significant differences were found between F and F+BNNT+US samples, while statistically significant differences were appreciated between F+BNNT+US and μG samples (p = 0.037) and also between μG and μG+BNNT+US samples (p = 0.035). Concerning peak amplitude, no differences were found between control, F and F+BNNT+US samples, while significantly higher peaks were measured on μG samples, in comparison with F+BNNT+US ones (p<0.01). μG and μG+BNNT+US also showed significant differences (p<0.01).

All the tested myotubes responded to caffeine and ACh, showing their ability to express functional ryanodine and nicotinic/muscarinic ACh receptors [53], [54], [55]. Such results demonstrated that muscle cells, co-cultured with fibroblasts, significantly benefit from both an anisotropically oriented topography and an intracellular BNNT-mediated electrical stimulation, not only in terms of gene and protein expression, but also in terms of electrical functionality.

### Conclusions

We developed engineered free-standing polyacrilamide gels provided with optimal mechanical features for skeletal muscle differentiation and with anisotropically oriented micro-grooves; we co-cultured normal dermal human fibroblasts and murine myoblasts on these substrates. We also supplemented the culture medium with boron nitride nanotubes, thus causing their internalization within cells, and we stimulated the co-culture with outer ultrasound sources. We found that boron nitride nanotubes were internalized only by the top cell layer, and localized inside early and late endosomes of muscle cells, while no particles were internalized by the underneath fibroblast layer. We also found that both micro-topography and nanoparticle-mediated stimulation deeply affected skeletal muscle differentiation at both gene and protein level, with the formation of longer, thicker and more functional myotubes on samples characterized by the synergy of topographical and nanotube-based stimuli. We also identified which human cytokines, produced by fibroblasts in the co-culture, were over-expressed in correspondence of surface micro-topography and BNNT+US-mediated stimulation during the muscle differentiation process. We envision that adding cholinergic neurons to the co-culture system could further improve myotube formation and maintenance, towards the realization of functional tissue engineered skeletal muscle for regenerative purposes (thus envisioning a translation of these findings from a mouse to a human cell model) or for the development of bio-hybrid actuators resembling the *in vivo* organization of animals’ natural muscle tissues, including neuromuscular junctions.

## Supporting Information

Table S1 **Summary of the most used abbreviations in the paper (in alphabetic order).**
(DOC)

Table S2 **List of the genes used as markers for skeletal muscle differentiation and corresponding primer sequences.** The sequences were designed to be specific for mouse cells (they do not detect human samples).
(DOC)

Table S3 **List of the genes used as markers for ECM protein production and corresponding primer sequences.** The sequences were designed to be specific for human cells (they do not detect mouse samples).
(DOC)
