# Supplementary material for: Boron Nitride Nanotube-Mediated Stimulation of Cell Co-Culture on Micro-Engineered Hydrogels
Source: PLoS One. 2013 Aug 14;8(8):e71707. doi: 10.1371/journal.pone.0071707 (PMC3743765; doi:10.1371/journal.pone.0071707)
Supplement: Table S1 — Summary of the most used abbreviations in the paper (in alphabetic order). (DOC) [file pone.0071707.s001.doc]

Table S1

| **Full name** | **Abbreviation** |
| --- | --- |
|  |  |
| α-actinin | Actn |
| Boron nitride nanotubes | BNNTs |
| Extracellular matrix | ECM |
| Flat samples | F |
| Flat samples provided with BNNTs and stimulated by ultrasounds | F+BNNT+US |
| Glycol chitosan | GC |
| Granulocyte colony-stimulating factor | G-CSF |
| Granulocyte/macrophage colony-stimulating factor | GM-CSF |
| Interferon γ | IFN-γ |
| Interleukin | IL |
| Macrophage inﬂammatory protein | MIP-1β |
| Micro-grooved samples | μG |
| Micro-grooved samples provided with BNNTs and stimulated by ultrasounds | μG+BNNT+US |
| Monocyte chemotactic protein | MCP-1 |
| Muscle LIM protein | MLP |
| Myosin heavy chain | MHC |
| Normal human dermal fibroblasts | nHDFs |
| Polyacrylamide | PA |
| Sarcomeric actin | Acta1 |
| Tumor necrosis factor | TNF-α |
| Ultrasounds | US |
|  |  |
