# Supplementary material for: Boron Nitride Nanotube-Mediated Stimulation of Cell Co-Culture on Micro-Engineered Hydrogels
Source: PLoS One. 2013 Aug 14;8(8):e71707. doi: 10.1371/journal.pone.0071707 (PMC3743765; doi:10.1371/journal.pone.0071707)
Supplement: Table S2 — List of the genes used as markers for skeletal muscle differentiation and corresponding primer sequences. The sequences were designed to be specific for mouse cells (they do not detect human samples). (DOC) [file pone.0071707.s002.doc]

Table S2

| **Gene** | **Abbreviation** | **Primer sequences** |
| --- | --- | --- |
| MyoD | MyoD | Sense:5’-GGCTACGACACCGCCTACTA-3’  Antisense: 5’-CTGGGTTCCCTGTTCTGTGT-3’ |
| Myogenin | Myogenin | Sense: 5’-TGTCTGTCAGGCTGGGTGTG-3’  Antisense: 5’-TCGCTGGGCTGGGTGTTAG-3’ |
| Muscle LIM Protein | MLP | Sense: 5’-TGGGTTTGGAGGGCTTAC-3’  Antisense: 5’-CACTGCTGTTGACTGATAGG-3’ |
| MRF4 | MRF4 | Sense: 5’-CGAAAGGAGGAGACTAAAG-3’  Antisense: 5’-CTGTAGACGCTCAATGTAG-3’ |
| α-actinin | Actn | Sense: 5’-TCATCCTCCGCTTCGCCATTC-3’  Antisense: 5’-CTTCAGCATCCAACATCTTAGG-3’ |
| Sarcomeric Actin | Acta1 | Sense: 5’-ATGGTAGGTATGGGTCAG-3’  Antisense: 5’-GATCTTCTCCATGTCGTC-3’ |
| MHC-IId-x | MYH1 | Sense: 5’-GCGACAGACACCTCCTTCAAG-3’  Antisense: 5’-TCCAGCCAGCCAGCGATG-3’ |
| MHC-IIa | MYH2 | Sense: 5’-GCAGAGACCGAGAAGGAG-3’  Antisense: 5’-CTTTCAAGAGGGACACCATC-3’ |
| MHC-IIb | MYH4 | Sense: 5’-GAAGGAGGGCATTGATTGG-3’  Antisense: 5’-TGAAGGAGGTGTCTGTCG-3’ |
| Perinatal MHC | MYH8 | Sense: 5’-ACTGAGGAAGACCGCAAGAA-3’  Antisense: 5’-CAGGTTGGCATTGGATTGTTC-3’ |
