# Supplementary material for: Boron Nitride Nanotube-Mediated Stimulation of Cell Co-Culture on Micro-Engineered Hydrogels
Source: PLoS One. 2013 Aug 14;8(8):e71707. doi: 10.1371/journal.pone.0071707 (PMC3743765; doi:10.1371/journal.pone.0071707)
Supplement: Table S3 — List of the genes used as markers for ECM protein production and corresponding primer sequences. The sequences were designed to be specific for human cells (they do not detect mouse samples). (DOC) [file pone.0071707.s003.doc]

Table S3

| **Gene** | **Abbreviation** | **Primer sequences** |
| --- | --- | --- |
| Fibronectin | FN | Sense:5’-GCGACTCTGACTGGCCTTAC-3’  Antisense: 5’-CCGTGTAAGGGTCAAAGCAT-3’ |
| Colagen type 1, alpha 1 | COL1A1 | Sense:5’-GCTGGTGTGATGGGATTC-3’  Antisense: 5’-GGGAACACCTCGCTCT-3’ |
| Collagen type 1, alpha 2 | COL1A2 | Sense:5’-GAAGTTGTTGATGGAGTTAATTTTTG-3’  Antisense: 5’-CCTCTTAAAATAACATTCCAAACCC-3’ |
| Collagen type 6, alpha 1 | COL6A1 | Sense:5’-TTGCACAGCACTAACAAGCC-3’  Antisense: 5’-TCACCTTCACACTGTCCACC-3’ |
